# Recent Trends in the Development and Application of Nano-Antioxidants for Skin-Related Disease

**DOI:** 10.3390/antiox14010027

**Published:** 2024-12-28

**Authors:** Yi Xue, Tao Wang, Ji-Peng Liu, Qi Chen, Xiao-Long Dai, Min Su, Yu-Hang Cheng, Cheng-Chao Chu, Yun-Qing Ren

**Affiliations:** 1 Department of Dermatology, The Children’s Hospital, Zhejiang University School of Medicine, National Clinical Research Center for Child Health, Hangzhou 310003, China; 2 Xiamen University Affiliated Xiamen Eye Center, Eye Institute of Xiamen University, School of Medicine, Xiamen University, Xiamen 361102, China; 3 Department of Dermatology, The Second Affiliated Hospital and Yuying Children’s Hospital of Wenzhou Medical University, Wenzhou 325027, China; 4 Department of Pharmacy, Xiamen Medical College, Xiamen 361023, China; sumin@xmmc.edu.cn

**Keywords:** skin, nano-antioxidants, ROS, nanomedicine

## Abstract

Skin is a vital barrier for the human body, protecting against external environmental influences and maintaining internal homeostasis. In addition, an imbalance of oxidative stress and antioxidant mechanisms can lead to skin-related diseases. Thus, for treating skin-related diseases, antioxidant therapy may be an important strategy to alleviate these symptoms. However, traditional drug therapies have limitations in treating these conditions, such as lack of lasting effect and insufficient skin permeability. Recently, nano-antioxidants, with their good permeability, sustained-release ability, multifunctionality, and other beneficial characteristics, have showed their advances in the exploration of skin-related diseases from research on safe therapies to clinical practice. Hereby, we review the latest research and advancements in nano-antioxidants for skin-related diseases. We categorize skin-related diseases into four main groups: skin inflammatory diseases, skin damage caused by ultraviolet rays, skin wound healing, and other skin-related conditions. Additionally, we summarize the prospects and potential future directions for nano-antioxidant drugs in treating skin-related diseases.

## 1. Introduction

In human tissues, the excessive accumulation or production of reactive oxygen species (ROS) can impair this defense mechanism, leading to related diseases [1]. As we all know, ROS encompass both oxygen radicals and non-radical oxidants and possess strong oxidizing abilities. Oxygen radicals encompass superoxide anion radicals (O_2_^·−^) and hydroxyl radicals (·OH). In contrast, non-radical oxidants include hydrogen peroxide (H_2_O_2_) and singlet oxygen (^1^O_2_) [1,2,3,4]. Meanwhile, the production of ROS is linked to various skin diseases [5]. For instance, in patients with atopic dermatitis and psoriasis, ROS-related markers are elevated [6]. In diabetic ulcers, the activity of iNOS is very low, and abnormal ROS levels increase the difficulty of treating skin ulcers [7]. Mitochondria produce the majority of ROS, and an excess can damage DNA, be cytotoxic, promote skin aging, and cause wrinkles and atypical pigmentation [8,9,10,11,12,13]. Skin damage caused by UV rays, such as photoaging and melasma, requires specific treatments. For skin aging, topical treatments include vitamin A and its derivatives, peptides (e.g., neurotransmitter inhibitory peptides), and antioxidants [14,15,16,17].

Until now, commercial products containing antioxidants have been promising treatments for skin-related diseases [18,19]. Currently, many antioxidants are in use and under research, and they can be roughly divided into two categories based on their characteristics. Among these, vitamins C and E and beta-carotene can be grouped together as they reduce oxidative stress by directly scavenging free radicals [13]. The second category consists of enzymes that scavenge oxygen free radicals, such as superoxide dismutase [20], catalase [21], and glutathione peroxidase [22]. However, local treatments with topical drugs often fail to meet clinical needs due to their limited drug penetration. Yang et al. [23] proposed the concept of “ROS science” by integrating three interdisciplinary research fields: the chemical mechanisms of ROS, their biological effects, and nanomaterials, aiming to better optimize treatment strategies. The long-term goal of “ROS science” is to design a new generation of nanomedicines that can produce or consume ROS to meet clinical needs by understanding ROS characteristics and integrating this knowledge with nanomaterials.

Recently, nanomedicine has developed rapidly in the past few decades, combining biomaterials with drugs to design nanomedicines with efficient delivery capabilities, providing new solutions for the treatment of skin-related diseases. Furthermore, many studies have introduced nanocarriers into the antioxidant treatment of skin-related diseases. Nanocarriers loaded with antioxidant drugs to exert synergistic effects are called “nano-antioxidants” (Table 1, Table 2 and Table 3). In this review, we summarize their roles in oxidative stress and skin-related diseases, and other conditions related to oxidative stress. We first classify skin-related diseases in detail, and elaborate on disease characteristics and the role of oxidative stress or ROS in the occurrence of diseases. Afterwards, we sort out the feasibility and current status of antioxidant therapy and discuss the important potential of nano-antioxidants as promising therapeutic targets for inflammatory skin diseases, skin photodamage, and wound healing. Finally, we summarize the limitations and advantages of the latest nano-antioxidant drugs and nanomaterial-based antioxidant therapies in the treatment of skin-related diseases and propose future development prospects.

## 2. Methods

A systematic literature search was conducted across five databases—Embase, Google Scholar, PubMed, Scopus, and Web of Science—to collect relevant studies published before November 2024. The search terms included “nano”, “antioxidants”, “psoriasis”, “atopic dermatitis”, “skin aging”, “melasma”, “skin wound healing”, “acne”, as well as the development and application of nano-antioxidants in skin-related diseases. This search aimed to gather information on the role of nano-antioxidants in treating skin disorders. Inclusion criteria: (1) studies evaluating the efficacy of nano-antioxidants in the treatment of skin-related diseases. (2) Research on the use of nano-antioxidants in combination with other drug delivery systems for antioxidant therapy. (3) Randomized controlled trials with consistent and reliable results. (4) In vitro studies investigating the application of antioxidant or nano drug delivery systems in skin conditions, wound healing, and specialized wounds. Exclusion criteria: (1) studies demonstrating the ineffectiveness of nano-antioxidants in treating skin-related diseases. (2) Articles with unclear or insufficient experimental design and results. (3) Non-randomized controlled trials. (4) Articles not published in English. (5) Reviews, book chapters, and editorials without original experimental data.

## 3. The Development of Nano-Antioxidants

**Figure 1:**
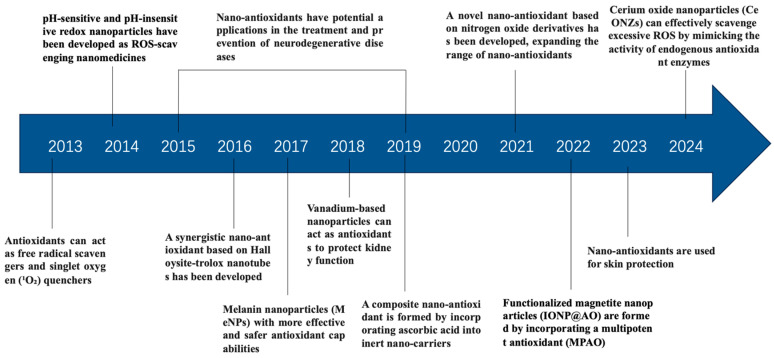
Schematic diagram of the development of nano-antioxidants over the past decade.

We conducted a search on the development of nano-antioxidants in the PubMed database and presented a timeline showcasing the key applications and conceptual developments that have significantly impacted the field over the past decade (Figure 1). Due to space limitations, many important findings and contributions were not included, and we apologize for this omission.

Nanoparticle antioxidants have emerged as effective agents for scavenging free radicals, quenching singlet oxygen (^1^O_2_), inactivating peroxides and other ROS, and acting as metal ion chelators and inhibitors of pro-oxidant enzymes [56]. To enhance therapeutic efficacy, two types of redox nanoparticles have been developed: pH-sensitive and pH-insensitive redox nanoparticles, designed to improve the treatment of oxidative stress-related diseases [57]. These nanoparticles have demonstrated potential in various applications, particularly in neurodegenerative disease therapies, where traditional antioxidants fail to cross the blood–brain barrier and prevent neuronal damage caused by oxidative stress [58]. In 2016, a synergistic nano-antioxidant, HNT-Trolox/Que, was developed by grafting Trolox on the surface of Halloysite nanotubes and loading quercetin in the lumen. This formulation exhibited superior antioxidant activity, particularly in inhibiting peroxyl and DPPH· radicals, due to the fast reaction of Trolox and sustained release of quercetin, showcasing the potential of synergistic nano-antioxidants in therapy [59]. Several other nanoparticle-based antioxidants have been introduced with promising medical applications. Melanin nanoparticles, containing functional groups such as catechols, amines, and imidazoles, offer a safer and more effective antioxidant therapy [60]. Additionally, molybdenum-based polyoxometalate nanoclusters have shown efficacy in scavenging ROS and alleviating acute kidney injury in mice, while the composite nano-antioxidant HNT/AH2, incorporating ascorbic acid, has enhanced the stability and activity of vitamin C [61,62]. The development of nitrogen oxide-derived nano-antioxidants in 2021 further expanded the range of available therapies [63]. Functionalized magnetite nanoparticles, incorporating multipotent antioxidants, have demonstrated enhanced antioxidant and antimicrobial properties, along with superparamagnetism, making them promising candidates for biomedical and nanomedicine applications [64]. Moreover, cerium oxide nanoparticles have been shown to mimic endogenous antioxidant enzymes, effectively scavenging ROS in cardiovascular diseases [65]. Recently, nano-antioxidants have also made significant strides in skin protection, where they have been used to reduce oxidative stress in inflammatory skin diseases and photoaging caused by UV exposure. These advancements signal a new era in skin protection and wound healing therapies [42].

## 4. Nano-Antioxidants for Skin Inflammatory Diseases

### 4.1. Psoriasis

Psoriasis is a non-contagious skin disease that causes pain, disfigurement, and disability. It has no cure and is linked to many other health issues, affecting over 60 million people worldwide [66,67]. The global prevalence of psoriasis ranges from 0.2% to 24.6%, while in Australia, the prevalence of adult psoriasis can reach up to 1.99% [68,69]. Among all types, plaque psoriasis is the most common (>80%) and typically presents as erythematous scaly plaques, which may also affect internal organs and joints, including areas such as the palms, soles, and nails [70]. The cause of psoriasis is complex and related to many factors. However, many studies have found that patients with psoriasis have elevated levels of ROS [71]. Karabovich et al. found that psoriasis patients experience oxidative stress, characterized by alterations in cellular components such as increased expression of proteasomes and immunoproteasomes, decreased mitochondrial immunoproteasome subunit expression, and elevated blood levels of 4-HNE [72]. Furthermore, the end products of malondialdehyde in red blood cells in serum or plasma are increased in patients with varying severities of psoriasis. There is conclusive evidence that oxidative stress is related to the pathogenesis of psoriasis [73,74]. In addition, TNF-α induces the production of ROS, which in turn produces cytokines to participate in the inflammatory response of the skin. It is worth mentioning that the activation of *NF-kB* is an important intermediate between TNF-α and ROS [75].

**Figure 2:**
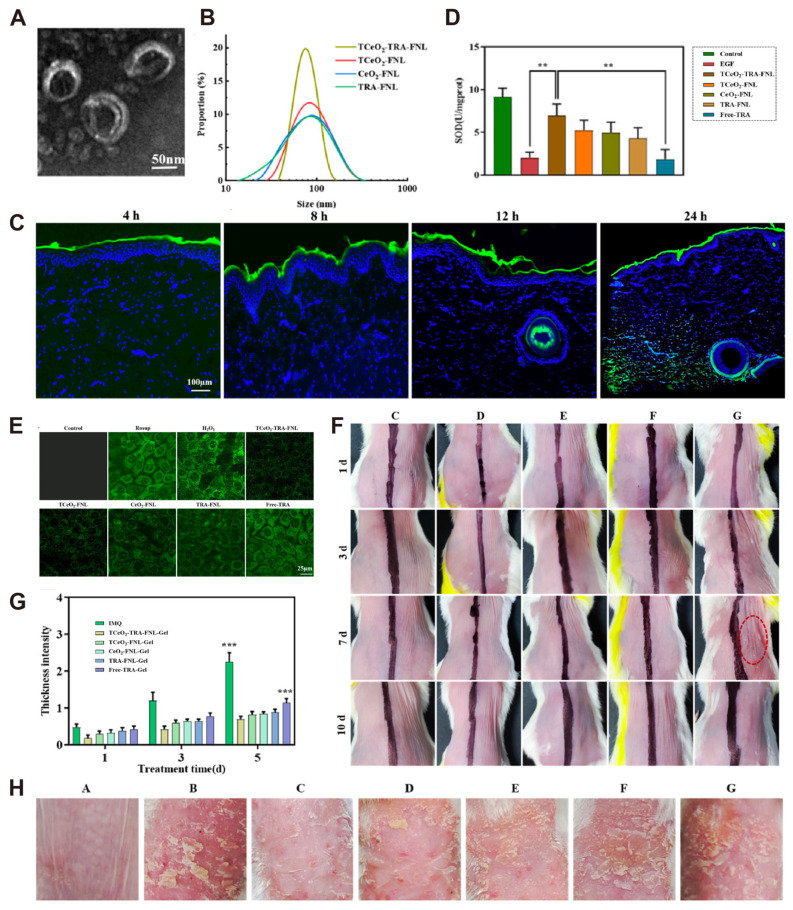
(**A**) TEM imaging results of TCeO_2_-FNL nanomaterials. (**B**) DLS measurement results of four groups of nanomaterials including TCeO_2_-TRA-FNL. (**C**) Penetration of flexible nanoliposomes in skin (4 h, 8 h, 12 h, 24 h). (**D**) HaCat cells were incubated with various nanomaterial groups for 4 h, followed by treatment with H_2_O_2_ for 24 h, after which SOD activity was measured. (**E**). Immunofluorescence was used to assess the intracellular ROS content of HaCat cells treated under the same conditions as depicted in the panel. (**F**) Results of skin irritation at different time points. (**G**) Skin thickness intensity scores of mice in different treatment groups at various time points in the psoriasis mouse model, along with typical images of treatment endpoints (**H**); ** *p* < 0.01, *** *p* < 0.001 [27]. CC BY 4.0.

Conventional topical treatments for psoriasis face challenges such as limited drug penetration and absorption. To address these shortcomings, innovative nanoformulations have been actively investigated in recent decades. Curcumin (CUR) is a widely studied bioactive polyphenol known for its antioxidant, anticancer, and anti-inflammatory potential. Algahtani et al. evaluated a combined CUR-Imiquimod(IMQ) (IMQ-CUR-nanoemugel) delivery system based on nanoemulgel and found that it could significantly reduce psoriasis-like skin adverse reactions in vivo, which illustrates that the use of CUR’s antioxidant properties combined with nanomedicine to treat psoriasis is of great significance [24,25,26]. Since cerium oxide(CeO_2_) nanoparticles possess strong antioxidant properties [35], Wang et al. [27] utilized the antioxidant properties of CeO_2_ combined with triphenylphosphine(TPP), preparing TCeO_2_. The nanoparticles were integrated into a nanodelivery system with gel, all-trans retinoic acid (TRA), and other components to create TCeO_2_-TRA-FNL-Gel, which effectively reduces inflammation and alleviates oxidative stress in HaCat cells. TEM photography was performed on TCeO_2_-TRA-FNL-Gel (Figure 2A), and DSL measurement revealed the particle size (Figure 2B). The skin penetration results suggested that the gel required 24 h to reach the dermis, likely due to its sustained-release behavior and high skin retention (Figure 2C). Additionally, the antioxidant capacity was evaluated by assessing the activity of the superoxide dismutase (SOD) enzyme. The H_2_O_2_ induction model demonstrated that TCeO_2_-TRA-FNL significantly increased SOD levels compared to other groups (Figure 2D). The green fluorescence intensity of TCeO_2_-TRA-FNL-incubated cells decreased significantly compared to other groups, indicating its excellent ability to scavenge ROS (Figure 2E). Skin irritation results at different time points showed that encapsulating TRA into liposomes reduced skin irritation, and TCeO_2_-TRA-FNL-Gel improved safety during use (Figure 2F). In the psoriasis mouse model, significant differences were observed in skin thickness (Figure 2G) and skin lesions (Figure 2H) between the TCeO_2_-TRA-FNL-Gel group and other groups. In conclusion, the TCeO_2_-TRA-FNL nanotransdermal drug system is effective, reduces oxidation, and can improve the symptoms of imizomod-induced mouse psoriasis. In addition, an efficacy evaluation of bilirubin nanoparticles (BRNP) in preclinical psoriasis models found that BRNP reduced ROS levels within keratinocytes and improved psoriasis symptoms [28]. In addition, Yao et al. [29] utilized the antioxidant properties of poly(ethylene glycol)-b-poly(propyl sulfide) (PEPS) to synthesize Deucravacitinib@PEPS gel, which has anti-inflammatory and antioxidant capabilities. This gel has been shown to reduce mitochondrial oxidative stress and attenuate skin inflammation in an IMQ-induced psoriasis model. In short, the aforementioned studies on the application of nano-antioxidant therapy in psoriasis have achieved significant therapeutic effects, offering promising new strategies for its treatment.

### 4.2. Atopic Dermatitis

**Figure 3:**
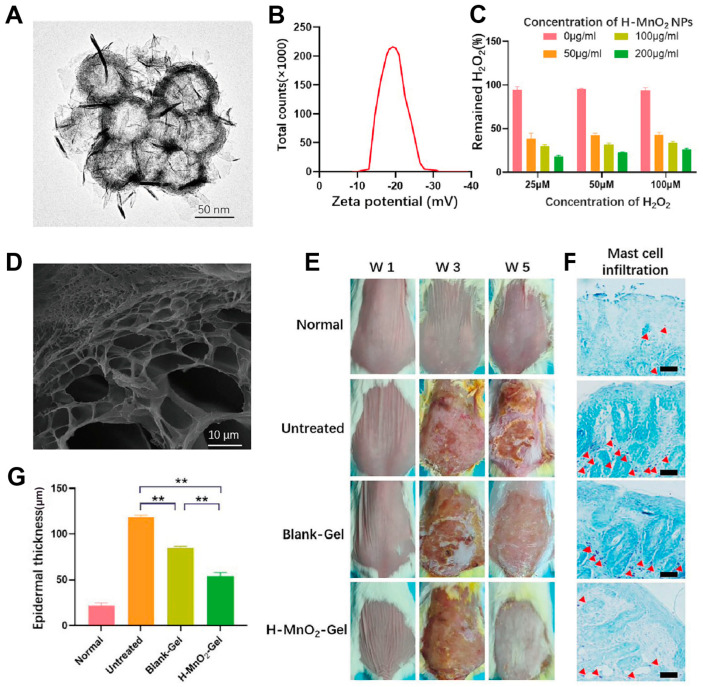
TEM (**A**) (scale bar = 50 nm) and potential (**B**) of H-MnO_2_ NPs. (**C**) After treating H_2_O_2_ with varying concentrations of H-MnO_2_ NPs, the remaining hydrogen peroxide was detected. (**D**) Cross-sectional SEM image of freeze-dried H-MnO_2_-Gel. (**E**) Representative images of the back skin of each group after treatment with different nanomedicines. (**F**) The results of skin tissue in each group at the end of treatment, and the number of the mast cells was measured (red arrowheads, scale bar = 100 μm). (**G**) Skin thickness in (**F**) was measured and statistically analyzed ** *p* < 0.01 [80]. CC BY 4.0.

Atopic dermatitis (AD) is a relapsing-remitting inflammatory skin disease commonly seen in children with an immune-mediated pathogenesis [76]. In 2019, at least 171 million people, accounting for 2.23% of the global population, were affected by AD, according to the Global Burden of Disease consortium [77]. Clinically, AD is characterized by dry skin, eczema, and severe itching, increasing the risk of asthma and allergies, thus placing a heavy burden on patients and society. It can also lead to significant social functioning changes and substantial direct and indirect costs. The pathophysiology of AD involves a complex interplay between dysfunctional epidermal barriers, skin microbiome abnormalities, and predominantly type 2 skewed immune dysregulation. These mechanisms can promote and interact with each other. For example, the weakness of the skin barrier caused by filaggrin deficiency promotes inflammation and T cell infiltration [78]. As early as two decades ago, studies reported that increased oxidative stress is related to the pathogenesis of atopic dermatitis. Recent research has found that excessive ROS production by mitochondria can lead to atopic dermatitis, possibly due to the inhibition of catalase [79]. In addition, oxidative stress in AD patients can damage keratinocytes, increase the expression of pro-inflammatory factors, promote local inflammation, enhance dermal inflammation, and reduce skin barrier function, suggesting that inhibition through antioxidant therapy may be a new direction in the treatment of AD. Qiu et al. [32] prepared ZIF-8 nanoparticles and developed a Zn-MOF hydrogel containing ZIF-8 nanoparticles (Gel@ZIF-8), which is a PVA-based hydrogel. The gel can remove ROS and has long-lasting antibacterial activity and cell biocompatibility. Gel@ZIF-8 significantly enhanced the therapeutic effect in AD-induced mouse models, likely by scavenging ROS and thereby regulating the inflammatory microenvironment to treat AD. Jia et al. [33] developed a hydrogel dressing incorporating dopamine nanoparticles, FAK inhibitors, and other components, which can alleviate symptoms in mouse AD models by scavenging ROS and inhibiting the FAK signaling pathway. This method targets AD-inducing factors, providing synergistic treatment to achieve optimal inflammation suppression and skin barrier repair. Kim et al. [34] developed an antioxidant hydrogel patch embedded with ceria nanoparticles, which resulted in a reduction of extracellular and intracellular ROS, including hydrogen peroxide, hydroxyl radicals, and superoxide anions, thereby reducing cellular damage caused by oxidative stress. Moreover, in AD animal experimental models, these hydrogel patches reduced levels of AD immune-related biomarkers such as mast cells and IgE antibodies, decreased mouse epidermis thickness, and improved the therapeutic effect on AD. Wu et al. [80] developed a hydrogel (H-MnO_2_-Gel) with antioxidant properties by incorporating hollow manganese dioxide(H-MnO_2_) nanoparticles, tannic acid (TA), and other nanocomponents. TEM visualization of H-MnO_2_ NPs is shown in Figure 3A, and DLS measurements revealed their size to be 49.4 ± 0.8 nm. Zeta potential results indicated a potential of −18.11 ± 1.01 mV (Figure 3B). To verify the ROS-scavenging ability of the nanomaterials, the H_2_O_2_ decomposition capacity of H-MnO_2_ NPs was evaluated in vitro. The results showed that the nanoparticles could effectively catalyze H_2_O_2_ (Figure 3C). It was found that 50 µg of H-MnO_2_ NPs could significantly reduce H_2_O_2_, indicating that H-MnO_2_ NPs can inhibit the production of ROS in AD. Subsequently, H-MnO_2_-Gel with antioxidant properties was successfully prepared through a simple physical cross-linking method and visualized by SEM (Figure 3C). The catalytically active solution of H-MnO_2_-Gel was tested in a simulated AD (20 µM H_2_O_2_) dermal tissue microenvironment and was found to effectively eliminate ROS generated within cells, indicating strong antioxidant ability. In the mouse AD model induced by DNCB, the therapeutic effect of the H-MnO_2_-Gel group was significantly better compared to other groups after continuous treatment for up to five weeks (Figure 3D). Histological examination revealed that the H-MnO_2_-Gel treatment group exhibited a significantly lower number of mast cells compared to the other two treatment groups (Figure 3F). Figure 3G shows the epidermal thickness between each group after the treatment, with the H-MnO_2_-Gel group being much lower than the other treatment groups and having statistical significance. These results indicate that H-MnO_2_ gel can improve the symptoms of AD and provide new evidence for the antioxidant treatment of AD.

We summarized the research related to AD, nanomedicines, and antioxidants, and found that hydrogels carrying nanomedicines with different functions and having antioxidant capabilities can significantly improve the symptoms of AD. These studies are expected to provide new strategies and theoretical foundations for the management of AD.

## 5. Nano-Antioxidants for UV-Radiation-Induced Skin Damage

### 5.1. Skin Aging

**Figure 4:**
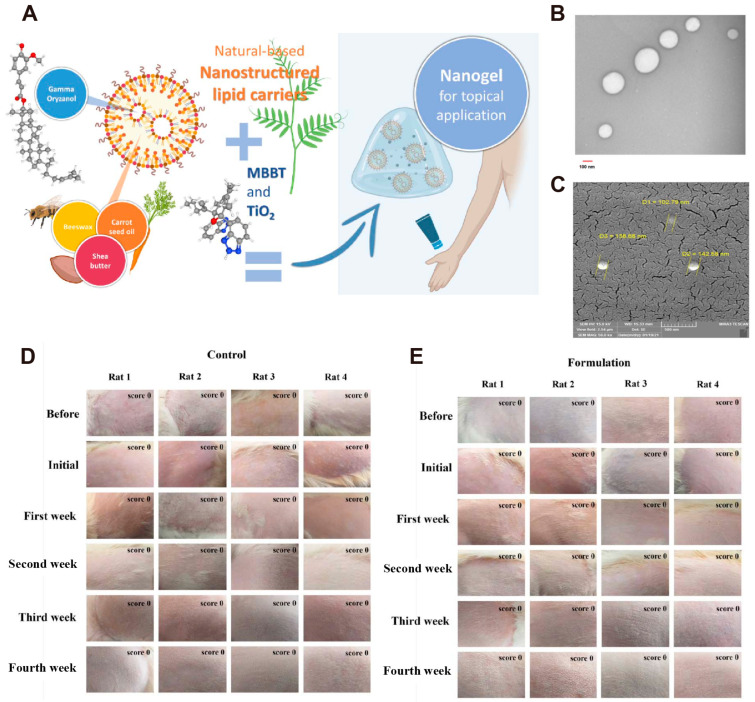
(**A**) The components of nanogel include Gamma oryzanol-Nanostructured lipid carriers, MBBT, and TiO_2_ (produced by Biorender). SEM (**B**) and TEM (**C**) images of nanostructured lipid carrier formulation loaded with gamma-oryzanol. The safety evaluation was conducted on albino rats using the Draize test, with the model rats treated with either physiological saline (**D**) or nanogel (**E**) [42]. CC BY 4.0.

Skin aging is generally caused by the accumulation of cellular damage and intrinsic aging from oxidative stress, also known as chronological aging. In addition, external environmental factors, such as ultraviolet (UV) radiation, poor nutrition, and air pollution, can also contribute to skin aging [81]. A study on skin aging in Asian individuals found that over 50% of cases exhibited a loss of skin elasticity, skin thinning, and the development of wrinkles in exposed areas [82]. This may be due to oxidative stress from accumulated ROS, which can damage lipids, proteins, nucleic acids, and organelles, leading to cellular senescence and skin aging [83]. ROS activates numerous signaling pathways, reduces collagen production, enhances matrix metalloproteinase (MMP) activation, and degrades connective tissue, ultimately accelerating skin aging [84]. To improve the solubility and local delivery of drugs, antioxidants can be combined with nanocarriers [85]. Therefore, incorporating antioxidants into nanoparticles can improve their effectiveness, inhibit oxidative stress, and promote DNA repair, ultimately aiding in skin repair. Avadhani et al. [38] designed a new type of nanotransfersomes based on epigallocatechin-3-gallate (EGCG), which can improve cell viability, reduce lipid peroxidation, inhibit the production of ROS in HaCaT cells, and suppress the expression of MMP-2 and MMP-9. In short, the nanotransfersomes have antioxidant and anti-aging effects. Lee et al. [86] studied (-)-catechin and found that it achieved anti-aging effects on the skin by clearing ROS accumulation. Additionally, (-)-catechin inhibited the expression of IL-1 and IL-6. The elastic highly permeated nanovesicles encapsulating L-Ascorbic acid-loaded spanlastics, designed by Elhabak et al. [39], can improve UV-induced skin damage, demonstrating their antioxidant protection and ability to prevent skin photodamage. Wang et al. [40] synthesized hyaluronic acid-ceramide (HA-CE) to modify nanoliposomes and prepared flexible nanoliposome-coated MITC with high stability, efficacy, and skin permeability. The aforementioned studies suggest that this nanoliposome can increase the activity of antioxidant enzymes and remove ROS in HaCaT cell experiments, indicating its potential to protect the skin. However, nanomaterials combined with antioxidants for the prevention or treatment of skin photoaging include not only nanovesicles and liposomes but also nanomaterials that can carry active peptides. Wang et al. [41] developed an innovative copper peptide nanocarrier that effectively reduces cellular inflammation, aging, and apoptosis induced by oxidative damage. This is achieved by regulating the PEG2/COX-2 and other related signaling pathways, thereby decelerating skin aging. Badalkhani et al. [42] developed a nanogel based on γ-oryzanol, which also contains TiO_2_ (an ultraviolet filter) and other nanoparticles. This nanogel (TiO_2_/MBBT nanogel) effectively filters ultraviolet rays and provides antioxidant benefits, thereby protecting the skin from damage. They demonstrated the synthesis process of the TiO_2_/MBBT nanogels (Figure 4A), performed SEM (Figure 4B) and TEM (Figure 4C) visualization of the nanolipid carrier, and found that it has a uniform amorphous spherical structure and high packaging efficiency. Additionally, the nanostructured lipid carrier’s particle size is around 149 nm. In the 4-week subacute Draize skin irritation test on rats (Figure 4D,E), the results showed that there was no skin irritation or allergy. This proves that the nanogel is safe for transdermal administration and provides natural antioxidant and sun protection benefits.

### 5.2. Melasma

**Figure 5:**
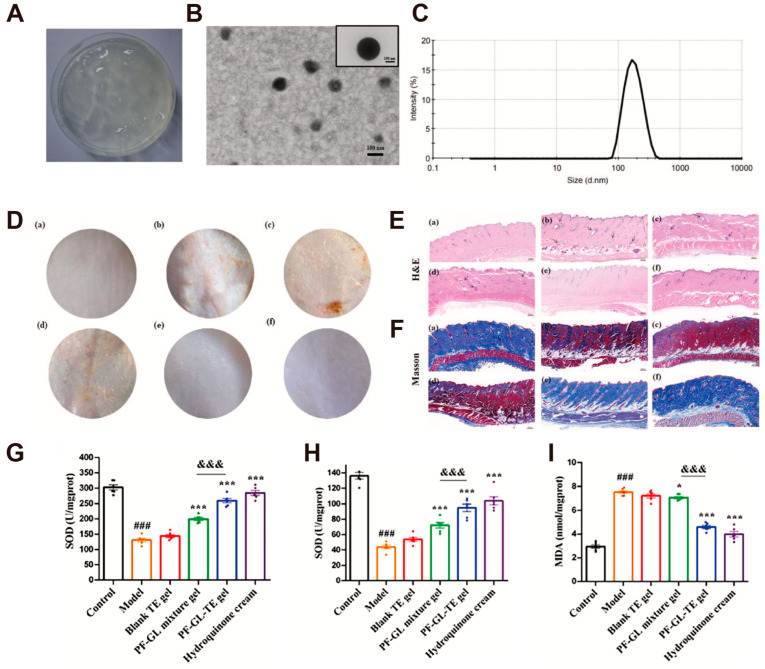
(**A**). Appearance of PF-GL-TE gel. TEM imaging results (**B**) and particle size (**C**) analysis of PF-GL-TE. (**D**) Typical skin images of rats in each treatment group. (**E**) HE-staining results of typical skin pictures of rats in each treatment group. (**F**) Masson staining results of typical skin pictures of rats in different group. The activity of SOD in rat liver (**G**) and skin (**H**) was detected, and the MDA content in rat skin (**I**) was also compared. ^###^ *p* < 0.001; * *p* < 0.05, *** *p* < 0.001; ^&&&^ *p* < 0.001 [46]. CC BY 4.0.

Melasma is a common, chronic, and refractory pigmentation disease that manifests as melanin deposits in sun-exposed areas [87]. Its prevalence varies by gender, skin type, and ethnicity, ranging from 1% to 50% [36]. Clinically, it typically presents as symmetrical reticular melanin pigmentation in three main areas: the central face, malar bones and jaw [88]. This pigmentation often imposes a severe burden on patients’ lives, affecting their social interactions, leisure activities, and emotional well-being. The pathogenesis of melasma is still unclear, some studies suggest it involves both exogenous and endogenous factors, such as basement membrane destruction, increased mast cells, and significant sun exposure [89]. Other studies indicate that genetic factors, cosmetics, ultraviolet radiation (UVB), pregnancy, hormone therapy, and phototoxic drugs may also contribute to its development [90]. Melasma treatment methods include topical drugs, lasers, and oral medications, with the most effective approach being a combination of hydroquinone, retinoic acid, and corticosteroids. However, laser or topical treatments alone often yield unsatisfactory results. Antioxidants including vitamin C, azelaic acid, cysteamine, carotenoids, and ellagic acid have achieved significant results in the clinical treatment of melasma [90]. Hence, among drug treatments, antioxidant drugs play an important role in treating melasma [16]. In recent years, with technological advancements, nanomedicines have become increasingly used in treating skin diseases. The rational integration of nanomaterials and antioxidant drugs can prolong drug efficacy, enhance transdermal properties, and improve overall treatment outcomes. Li et al. [45] developed a nanomedicine based on ascorbyl palmitate-transfersomes (AP-TF), which encapsulates AP into transfer bodies (TF) to achieve slow release. In the model, AP-TF demonstrated a better anti-chloasma effect than free AP and improved oxidative stress and inflammation in the skin. Additionally, this study suggests that developing new nanocarriers and improving drug delivery efficiency can enhance chloasma treatment, providing a new strategy for managing this condition. Due to the low stability of ascorbic acid (vitamin C) in cosmetic formulations, scientists developed a stable compound called magnesium ascorbyl phosphate (MAP) to enhance its stability. Kandil et al. [91] designed a localized nanoscale ethanolosome and liposome delivery system by integrating MAP. The ex vivo MAP penetration percentage of ethanolosomal and liposomal gels was lower compared to other ethanolosome and liposome preparations, but skin retention was higher (after 8 h). Clinically evaluated for the treatment of melasma, the MAP ethanolosome significantly improved symptoms with minor side effects. In addition, Xiao et al. [46] developed PF-GL-TE gel based on Paeoniflorin (PF) and glycyrrhizic acid (GL). Since PF and GL have anti-inflammatory, antioxidant, and inhibition of melanin formation, PF-GL-TE gel, the gel itself has high transdermal permeability, thus improving its anti-chloasma effect. Xiao et al. successfully prepared PE-GL-TE-gel (Figure 5A) and performed TEM (Figure 5B) visualization and particle size measurement of PE-GL-TE, and found that it was a small, uniform, and average particle size 167.9 ± 4.7 nm (Figure 5C). A chloasma rat model was then established using progesterone injection and UVB irradiation. The model was treated with gel or various preparations, and the treatment outcomes were observed. The typical skin of rats in different treatment groups was photographed (Figure 5D), HE-stained (Figure 5E), and Masson stained (Figure 5F). After 30 days of treatment, the results showed that compared with other groups, the PF-GL-TE gel group had no obvious pigmentation (Figure 5D), and the skin was smooth and moist. HE-staining results indicated inflammatory cell infiltration in the chloasma model group, while the treatment group (PF-GL-TE gel) exhibited a marked reduction in such infiltration (Figure 5E). Masson’s trichrome staining revealed a significant reduction in collagen in the model group, while the PF-GL-TE gel group showed a marked restoration in collagen content and density (Figure 5F). Additionally, statistical analysis was performed on SOD activity and malondialdehyde (MDA) levels in the skin of each treatment group (Figure 5G–I). In summary, these studies underscore the significance of antioxidant therapy and offer new strategies for treating melasma.

## 6. Nano-Antioxidants for Skin Wound Healing

### 6.1. General Wound Healing

**Figure 6:**
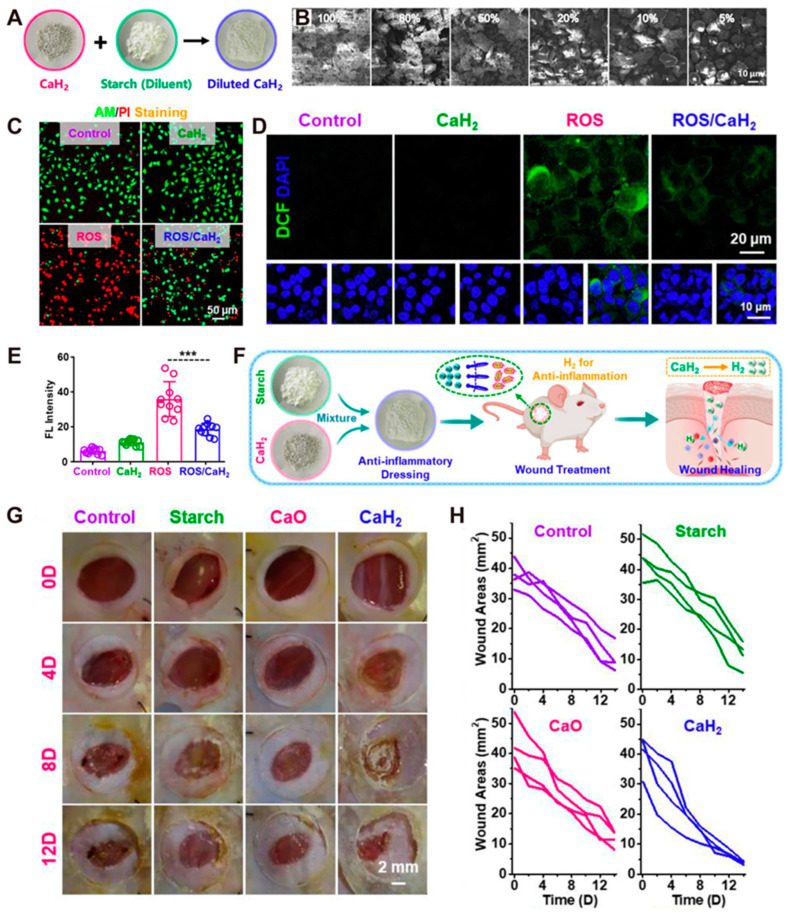
(**A**) Procedure for preparation of CaH2 Dressing. (**B**) SEM images of CaH2 dressings with different CaH_2_ contents. (**C**) Confocal images of HUVEC cells with different treatment groups (ROS, CaH_2_, ROS/CaH_2_, and the control group). (**D**) HUVEC cells were treated as in (**C**), followed by staining with DCFH-DA and confocal visualization. (**E**) Statistical analysis of DCF levels in different groups of cells in (**D**) (n = 10). (**F**) Flow chart of CaH_2_ dressing in the treatment of wound healing in this study. (**G**) Photos of skin wound healing at various time points (0, 4, 8, 12 days) post-treatment in different treatment groups. (**H**) Wound healing curve of healthy mice in (**G**) *** *p* < 0.001 [47]. Copyright 2022 Wiley-VCH GmbH.

Surgery, serious accidents, burns, and other injuries can all threaten skin health. Skin wounds pose a significant economic burden globally (USD 28.1 billion–USD96.8 billion) [92]. Wound healing generally involves inflammatory, epithelial, proliferative, and maturation phases. Delayed wound healing is a common complication after surgery; if the inflammation phase is prolonged or the wound remains in a pro-inflammatory state, healing will be delayed [93]. Moderate levels of ROS are crucial for body metabolism and wound healing, but excessive ROS in the wound microenvironment can damage immune cells, suppress inflammatory responses, and induce a strong inflammatory reaction, thereby hindering wound healing. Balancing ROS in wounds requires further research, potentially integrating enzymes or materials that effectively remove ROS to design nanomedicines or dressings for precise treatment [94]. A calcium hydride (CaH_2_) dressing has shown efficient ROS-scavenging ability. The CaH_2_ dressing releases hydrogen, effectively reducing inflammation levels, inhibiting the secretion of pro-inflammatory cytokines, enhancing the infiltration of anti-inflammatory immune cells, and promoting the generation of new blood vessels and collagen, thereby accelerating the wound healing process. Gong et al. [47] developed a CaH_2_ dressing by combining starch and CaH_2_ (Figure 6A), and used SEM to analyze powders with different CaH_2_ contents (100%, 80%, 50%, 20%, and 10%) for visualization (Figure 6B). Calcein-AM/propidium iodide staining of HUVEC cells treated with CaH_2_, ROS, and ROS/CaH_2_ revealed that almost all cells in the ROS-treated group were dead (red signal), while all cells in the CaH_2_ group were living (green signal) (Figure 6C). Afterwards, intracellular ROS levels were detected, and the results showed that there was almost no green signal in the CaH_2_ group, indicating that ROS levels were very low (Figure 6D). The ROS group exhibited a strong green signal, which was statistically significant compared to the ROS/CaH_2_ group (Figure 6E). CaH_2_ dressing has been shown to promote wound healing in healthy mice (Figure 6F). Additionally, the wound closure rates of mice treated with CaH_2_ dressing, control group, starch group, and CaO group (8 days post-treatment) were approximately 65%, 37%, 36%, and 43%, respectively, demonstrating that the CaH_2_ dressing promotes wound healing and removes ROS more effectively than other groups (Figure 6G,H). Mao et al. [48] combined selenium nanoparticles (SeNPs), known for their antioxidant and anti-inflammatory properties, with highly permeable bacterial cellulose (BC) to create a multifunctional BC/Gel/SeNPs hydrogel. This hydrogel not only exhibits a slow release of its components but also possesses excellent anti-inflammatory and antioxidant properties. It promotes wound healing, reduces inflammatory reactions, and prevents wound infection in a rat wound model (full-thickness skin). He et al. [49] developed a nanozyme hydrogel (Cu_2_Se/F127) with SOD enzymatic activity that promotes fibroblast migration and enhances wound healing in a mouse acute wound model, likely due to the antioxidant properties of Cu_2_Se. The hydrogel significantly promoted collagen deposition on the 7th day of treatment compared with other groups, without affecting the weight of the mice. CUR, the bioactive component of turmeric, has several properties including anti-inflammatory and antioxidant properties, and can increase the production of fibroblasts and accelerate wound contraction and epithelialization. Therefore, Li et al. [50] developed an EGF-modified spray by rationally integrating CUR-loaded chitosan nanoparticles with epidermal growth factor (EGF), which effectively promotes wound healing, likely due to the antioxidant properties of CUR. In summary, the aforementioned studies have preliminarily confirmed the crucial role of antioxidant treatment in wound healing.

### 6.2. Special Wound Healing

**Figure 7:**
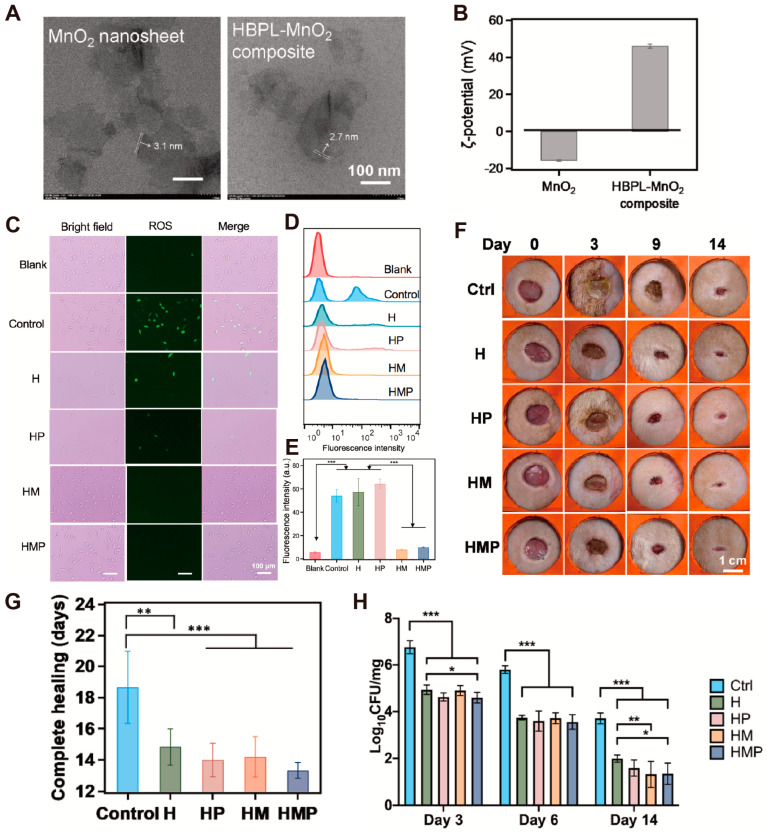
TEM visualization pictures (**A**) and Zeta potential (**B**) of HBPL-MnO_2_ and MnO_2_ nanosheets. The antioxidative stress performance (expression of ROS) of L929 fibroblasts and various hydrogel groups was tested (**C**). Fluorescence intensity (ROS intensity) detected by flow cytometry (**D**) and statistical analysis (**E**). (**F**) The diabetic model (MRSA infection) was treated with different gels, and representative pictures were taken at various time points. (**G**) The time required for complete wound healing in different treatment groups. (**H**) The bacterial colony density in infected skin tissue was quantified for each treatment; * *p* < 0.05, ** *p* < 0.01, *** *p* < 0.001 [53]. Copyright © 2022 Elsevier Ltd.

Diabetes, a widespread chronic disease caused by insulin resistance or deficiency, is rising. The global prevalence of diabetes in 2019 was 463 million, with the number of people expected to increase to 578 million by 2030 and 700 million by 2045 [95]. Furthermore, diabetic patients face a high lifetime risk (15–34%) of foot ulcers, which are prone to recurrence and infection [96]. The recurrence rate of traditional therapy for treating diabetic ulcers exceeds 70%, requiring expensive and prolonged treatment, which places a significant burden on patients [97]. Diabetic wound healing often deviates from the four stages of normal wound healing and typically requires medications or other treatments to promote healing due to the complex local environment characterized by hypoxia, elevated reactive oxygen species, and high Ph [98]. For healthy individuals, wound healing requires little external intervention. However, in diabetic patients, the healing process of foot ulcer wounds resembles that of normal acute wounds but often stagnates at certain stages [99]. As mentioned, the complex and infection-prone microenvironment of diabetic wounds, combined with the heavy economic burden of diagnosis and treatment, underscores the urgent need to explore new treatment methods and technologies [100]. The current clinical treatment of diabetic wounds includes surgical debridement, pressure offloading, antibiotic treatment, and wound dressing [101]. Non-surgical debridement agents can be useful when surgical debridement is ineffective. Currently, numerous studies are exploring the use of antioxidants combined with nanomaterials for diabetic wound healing, potentially offering new strategies for future treatments. Li et al. [51]. designed a non-enzymatic antioxidant MXene nanosheet hydrogel that releases oxygen repeatedly to create an improved oxidative environment for diabetic wounds, promoting healing. Additionally, this hydrogel is injectable, adheres to tissues, has hemostatic properties, and crucially, exhibits antioxidant functions. Additionally, Zhai et al. [52] developed a multifunctional hydrogel incorporating O-nitrobenzene (NB) and ZnO nanoparticles. The NB in the hydrogel effectively captures oxygen free radicals, exhibiting significant antioxidant capabilities and inhibiting H_2_O_2_ by more than 50% within 8 h, thereby promoting diabetic wound healing. Tu et al. [53] developed a multifunctional nanozyme hydrogel with antibacterial properties, ROS-scavenging capabilities, and NO-promoted drug release to promote wound healing (diabetic). TEM imaging was used to visualize MnO_2_ nanosheets and HBPL-MnO_2_ nanosheet composites (Figure 7A). The potential of MnO_2_ nanosheets and HBPL-MnO_2_ nanosheet composites was also measured, showing a change in potential from −15.7 mV to +46.2 mV after HBP adsorption (Figure 7B). Next, the antioxidative stress abilities of various hydrogel groups were tested by adding H_2_O_2_ to L929 cells, which were then co-incubated with the different hydrogel groups. Subsequently, immunofluorescence was used to detect intracellular ROS levels, revealing almost no green fluorescence in the HMP group (Figure 7C), which indicates low ROS levels. This finding was further confirmed by flow cytometry analysis, showing minimal fluorescence intensity (Figure 7D), corroborating the reduced ROS presence in the HMP group. Statistical analysis of the fluorescence intensity (Figure 7E) demonstrated that the HMP group’s fluorescence intensity was significantly lower than that of the control group. The therapeutic effect of HMP hydrogel was later verified in a diabetic wound model (MRSA infection), and the results showed it effectively promotes wound healing (Figure 7F,G). Additionally, after 14 days of treatment, the remaining bacterial colonies in the skin tissue were counted, revealing that the HMP hydrogel significantly reduced MRSA bacteria in the body (Figure 7H).

**Figure 8:**
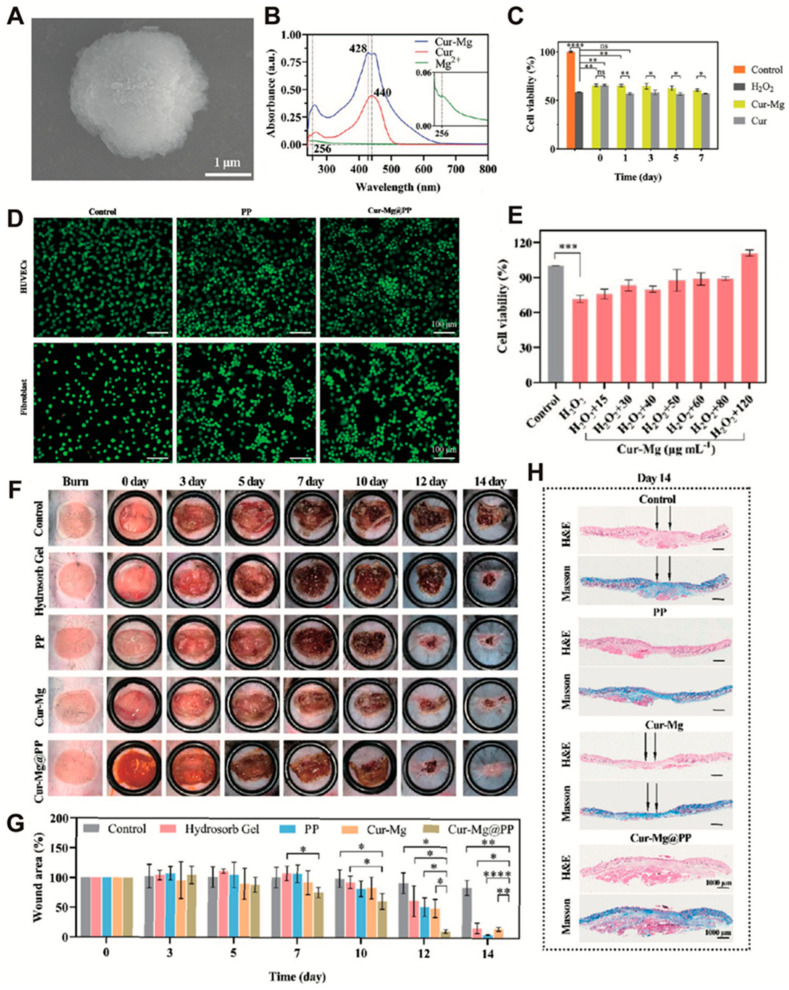
(**A**) Field emission-SEM visualization image of Cur-Mg. (**B**) Absorption spectrum of the main material (or component). (**C**) Evaluation of antioxidant effects of Cur and Cur-Mg at different time points. (**D**) Immunofluorescence images of fibroblasts and HUVEC cells co-cultured (72 h) with different hydrogel groups (live/dead double staining). (**E**) Results on cell viability: effect of varying concentrations of Cur-Mg on HUVEC (co-cultured with or without H_2_O_2_). (**F**) The infected burn wounds treated with various hydrogel (group) treatments. (**G**) The area in each group was evaluated (n = 3). (**H**). HE and Masson trichrome staining images of the different groups after 14 days of burn wound treatment; * *p* < 0.05, ** *p* < 0.01, *** *p* < 0.001, **** *p* < 0.0001 [55]. Copyright © 2023 American Chemical Society.

In addition, approximately 300,000 people were killed by fire-related burns every year and most deaths (95%) were observed in low- and middle-income countries [102]. The treatment of burn-related wounds (concurrent infections or other complications) also imposes heavy consequences on patients and society [103]. After severe burns, a large number of inflammatory mediators, including reactive nitrogen species and ROS, were released. ROS was associated with immunosuppression, sepsis, and tissue damage [104]. Therefore, the development of peroxisomes, antioxidants nanomedicines, or dressings may promote wound healing. β-carotene has excellent antioxidant activity. Darban et al. [105] developed a nanohydrogel system based on β-carotene, polyglyceryl stearate, graphene oxide, and gelatin, which has antioxidant and antibacterial effects, good biocompatibility, and sustained release of β-carotene. This nanohydrogel promotes the immune response and enhances burn wound healing by releasing β-carotene to increase the number of monocytes and macrophages. In addition, Huang et al. [54] developed an antibacterial hydrogel (PPY@PDA) with antioxidant properties, effective for burn wound treatment, adding new evidence that antioxidant therapy promotes wound healing. Moreover, Gong et al. [55] developed a novel antioxidant hydrogel by integrating curcumin, γ-glutamic acid, and other components. This hydrogel is primarily based on Cur-Mg particles, with SEM images showing the main nanoparticles (diameter: 5 μm) (Figure 8A). The absorption spectra of the main components in the material were measured, and the results showed that Cur and Cur-Mg have absorption peaks at 440 nm and 428 nm, respectively (Figure 8B). Furthermore, the antioxidant capacity of Cur-Mg was tested, revealing that Cur-Mg has stronger antioxidant capacity than other groups (Figure 8C). As shown in the figure, cells were treated with different treatment group to test the biocompatibility of the different hydrogels (72 h). Immunofluorescence was used to demonstrate the live and dead cell staining (Figure 8D). To evaluate the antioxidant effect of Cur-Mg nanoparticles, the viability of HUVECs was assessed using CCK-8 after co-incubation with Cur-Mg and H_2_O_2_ (Figure 8E). In addition, a mouse burn-infected wound model (Figure 8F) was established to explore the wound treatment and antibacterial capabilities of different hydrogel treatment groups. At the end of treatment (14th day), the wound area was statistically analyzed (Figure 8G). Afterwards, the skin tissue from each treatment group was removed and stained with HE and Masson to compare epithelialization and collagen deposition (Figure 8H).

In addition to diabetic and burn wounds, there are also related studies on the antioxidant properties of nanomedicines in healing radiation-damaged wounds. Wound healing in radiation-induced skin injuries is a significant clinical challenge. Zhou et al. [106] designed a nanoparticle-based nanocomposite using mesoporous silica (MS), cerium (IV) oxide (CeO_2_), and miR129. This MS-CeO_2_-miR129 nanocomposite has notable anti-ROS, anti-HIF-1α, and anti-radiation properties. It promotes angiogenesis and collagen deposition, thereby enhancing wound healing in a mouse model of radiation-induced skin damage. In short, many studies focus on the application of nanomedicines combined with antioxidants for wounds with special injuries. By using nanomedicines to rationally deliver antioxidants to the damaged site, excess ROS can be removed, promoting wound healing. This approach represents an important research direction for the future. These studies provide new strategies and research directions for healing special wounds.

## 7. Others

In addition to their applications in dermatitis and wound healing, antioxidants also play an important role in bacterial infectious diseases. Acne, a chronic inflammatory disease, is caused by androgen-induced sebum increase, changes in keratinization, inflammation, and colonization by *Propionibacterium acnes*. More than 20% of teenagers have facial scars caused by acne [107]. The pathogenesis of acne involves increased sebum production, follicular hyperkeratosis, bacterial colonization, and an inflammatory response. The acne affects the emotional and mental health of adolescents, potentially leading to low self-esteem, depression, and anxiety [108]. Moderate acne can be treated with a combination of topical therapies, such as benzoyl peroxide, retinoids, and antibiotics. However, severe acne requires further exploration of new treatment methods due to the side effects associated with current medications. Due to their small diameter, nanoparticles can more easily penetrate the skin barrier and reach deeper layers of the skin [109]. Thymol (TH) possesses antibacterial, antioxidant, and antiseptic properties. It can regulate the stratum corneum barrier function and restore skin homeostasis by scavenging oxygen free radicals [110]. Therefore, Folle et al. [111]. developed a nanoparticle based on TH and Poly(lactic-co-glycolic) acid (PLGA) to treat skin acne infection. These TH-NP nanoparticles, with an average diameter of less than 200 nm, exhibit antibacterial activity (inhibiting *Propionibacterium acnes*) and antioxidant effects, and can slowly release the natural active compounds of TH. Ivanova et al. [112]. used self-assembled nanocapsule technology to develop an essential-oil-loaded nanocapsule, which can “intelligently” release active ingredients in environments conducive to acne proliferation, inhibit *Propionibacterium acnes*, and remove ROS to prevent damage to human skin cells. Abdel-Monem R, et al. [92] designed a Cur-FA-MM nanogel based on nanogel mixed micelles (MM), cur, and fusidic acid (FA). Cur-FA-MM nanogel can achieve active antioxidant and antibacterial effects. The application of antioxidants in other diseases, such as the chemoprevention of skin cancer, involves using cationic ultraflexible nanocarriers to enhance the local delivery of highly lipophilic antioxidant diindolylmethane derivatives, inhibiting UV-induced DNA damage and skin carcinogenesis [113]. In short, nanomedicines with antioxidant effects can eliminate excessive ROS in acne and other skin diseases, thereby achieving therapeutic outcomes and providing a new strategy for treating infectious skin diseases.

## 8. Conclusions and Future Prospects

Antioxidant activity refers to the ability of a substance to delay or inhibit oxidative reactions by neutralizing free radicals, suppressing lipid peroxidation, or reducing oxidative stress. Common methods for assessing antioxidant activity include the 2,2-diphenyl-1-picrylhydrazyl (DPPH) radical-scavenging assay, the 2,2′-azino-bis(3-ethylbenzothiazoline-6-sulfonic acid) (ABTS) radical-scavenging assay, the oxygen radical absorbance capacity (ORAC) assay, and the ferric reducing antioxidant power (FRAP) assay. Antioxidants play a crucial role in disease prevention and treatment, but the lack of standardized evaluation criteria limits their research and clinical application. Establishing a multidimensional evaluation system should encompass chemical activity, intra- and extracellular effects, and disease-specific biomarkers, with a particular focus on the biocompatibility and targeting ability of nano-antioxidants. The adoption of unified international standards will enhance the consistency of antioxidant research and its translational potential. The traditional methods of delivering drugs to the skin are often ineffective due to the skin’s permeability and the limited antioxidant capacity of these drugs. This results in the inability of the drug to effectively reach the skin lesion, necessitating repeated treatments or multiple administrations. This not only poses significant challenges for treating skin-related diseases but also imposes a heavy burden on patients and society, including economic and psychological strains, which warrants the attention of scientists. Researchers are actively exploring new nano-antioxidant drugs for skin-related diseases to address these challenges. These new preparations offer the advantages of traditional nanodrugs, such as good permeability and antioxidant capacity, along with enhanced therapeutic functions, such as strong delivery capabilities and improved efficacy. This could potentially overcome the current shortcomings in skin disease treatments and bring new hope to patients. New nano-antioxidants represent a significant breakthrough in treating skin-related diseases. They leverage the unique properties of nanomaterials to maximize drug efficacy, achieving synergistic effects. For example, some nanomaterials, such as carbon nanomaterials, cerium oxide nanoparticles, and manganese dioxide particles, possess inherent antioxidant properties [114]. These materials can load drugs while simultaneously providing antioxidant effects. By incorporating antioxidant nanomaterials, the reliance on traditional antioxidant drugs can be reduced. Additionally, skin tissue produces a large amount of ROS when stimulated by external factors, making the removal of excess ROS crucial for maintaining internal homeostasis and treating diseases. Therefore, new nano-antioxidants are poised to play a significant role in skin-related diseases and wound healing, offering unprecedented application possibilities. Moreover, numerous studies have reported on nanozyme antioxidants, which could potentially replace traditional antioxidants. Clinical trials are anticipated in the future, potentially expanding the antioxidant market.

While nano drug delivery systems offer promising solutions for skin-related diseases, they still face significant challenges such as limited stability, low drug loading capacity, and concerns regarding long-term safety and efficacy. Addressing these limitations requires ongoing optimization and innovation. However, with continuous advancements in the design and application of nano-antioxidants, these challenges are gradually being mitigated, paving the way for more effective treatments. We have identified three key advantages of nano-antioxidants: (1) enhanced permeability, allowing them to penetrate deep into the skin layers and target underlying pathological processes; (2) sustained-release properties, which extend the duration of therapeutic action and reduce the frequency of dosing; and (3) multifunctionality, which enables their integration with various therapeutic modalities such as photodynamic therapy, sonodynamic therapy, and gene therapy. This versatility not only enhances the antioxidant capacity but also allows for a more comprehensive approach to treatment, where a single therapeutic platform can address multiple aspects of a disease. In this review, we have detailed the progress of nano-antioxidant drugs in treating a range of skin conditions, including atopic dermatitis, psoriasis, skin aging, melasma, general wound healing, diabetic wounds, burn wounds, and acne. The evidence suggests that nano-antioxidants can significantly improve outcomes in these conditions. However, it is crucial to recognize that oxidative stress manifests differently across these diseases, necessitating personalized treatment strategies to optimize therapeutic efficacy. In addition, the potential of nano-antioxidant drugs in dermatology is vast, but their successful clinical translation will depend on overcoming existing hurdles such as enhancing stability, improving loading capacities, and ensuring biocompatibility. Future research should focus on refining these nanoformulations to create more robust and reliable therapeutic agents. Moreover, clinical trials should be prioritized to validate the efficacy and safety of these treatments in diverse patient populations. Ultimately, the goal is to transition from promising preclinical findings to practical, widely accessible therapies that can alleviate the burden of skin-related diseases on patients and healthcare systems. By advancing the development and application of nano-antioxidants, we can move closer to achieving personalized, effective, and sustainable treatment solutions for a broad spectrum of dermatological conditions.

## Figures and Tables

**Table 1:** A brief summary of skin inflammatory diseases treatment based on nano-antioxidants strategies.

Diseases	Active Ingredient	Nanomaterial	Benefit	Ref.
Psoriasis	Curcumin, Imiquimod (IMQ)	Nanoemulgel (NEG)	Reduced the psoriasis-like skin reaction	[24]
	Epigallocatechin gallate	Microneedle	Improved treatment outcomes in both psoriasis-like and prophylactic psoriasis-like animal models	[25]
	Babchi oil	Cylodextin-based nanocarriers	Anti-psoriasis activity and antioxidant stress	[26]
	TCeO_2_	TCeO_2_-TRA-FNL-Gel	greatly alleviate the psoriasis symptoms	[27]
	Bilirubin	Polymer polyethylene glycol	Ameliorating psoriasis-like skin inflammation	[28]
	Deucravacitinib	Polyethylene glycol block-polypropylene sulphide	Inhibited STAT3 signaling; alleviated ROS generation	[29]
	Platinum (Pt)	Ultrathin Pt nanowires (NWs)	Significantly attenuated psoriasis-like skin inflammation	[30]
	Methotrexate (MTX), chlorine e6 (Ce6)	HA-modified hollow mesoporous organosilica nanoparticles (HMN)	Markedly enhanced the therapeutic effectiveness against IMQ-induced psoriatic-like skin inflammation in vivo	[31]
AD	PVA-based hydrogel	Zn-MOF	Scavenging ROS; reduce the thickness of epidermis	[32]
	Polydopamine nanoparticles (PDA NPs)	HCPF hydrogels	This gel effectively reduces inflammation, alleviates oxidative damage, and repairs the epidermal barrier in atopic dermatitis (AD) skin	[33]
	Ceria (Ce) nanoparticles	CeNP hydrogel	Reduce intracellular and intracellular ROS, reduce epidermal thickness and AD-related immune indicators in mouse AD models	[34]
	Ammonium chitosan, tannic acid, hollow manganese dioxide (H-MnO_2_)	H-MnO_2_-Gel	Clearing ROS and regulating the inflammatory microenvironment	[35]
	Cetirizine hydrochloride-loaded Prussian blue NPs (CET@PB NPs), living bacteria of Bacillus subtilis (B. subtilis, Bs)	Microneedle	Demonstrate superior antibacterial and anti-inflammatory properties the MN patches	[36]
	Rh Co	Mated-atom nanozyme (MAN)	Successfully accelerated the recovery of eczema in mice over time	[37]

**Table 2:** A brief summary of UV-radiation-induced skin damage treatment based on nano-antioxidant strategies.

Diseases	Active Ingredient	Nanomaterial	Benefit	Ref.
skin aging	Epigallocatechin-3-gallate	Hyaluronic-acid-loaded nanotransfersomes	Reduced lipid peroxidation, intracellular ROS, and MMP expression in HaCaT cells	[38]
	l-Ascorbic acid	Spanlastics	Provides antioxidant protection against skin photodamage	[39]
	Moringa oleifera seed isothiocyanate	Flexible nanoliposomes	Boosts antioxidant enzyme activity, scavenges ROS, protects skin	[40]
	Copper peptide (GHK-Cu)	Rigid-flexible nanocarriers	against oxidative damage, anti-inflammatory	[41]
	γ-oryzanol	Nanostructured Lipid Carriers and TiO_2_/MBBT	Skin photoprotective properties and compatibility	[42]
	Polydopamine (PDA)	Nanoparticles (NPs)	A significant increase in the hair density of DOX-treated mice following UPDA NPs treatment	[43]
	Nanovesicles isolated from the juice of Citrus limon (LNVs)	Extracellular vesicles (EVs)	LNV pre-treatment counteracted the ROS increment caused by H_2_O_2_ treatment or UVB irradiation	[44]
melasma	Ascorbic palmitate	Transfersomes	Relief of oxidative stress and inflammation	[45]
	Paeoniflorin, glycyrrhizic acid	PF-GL-TE	Increases SOD activity and reduces skin oxidative damage	[46]

**Table 3:** A brief summary of skin wound healing based on nano-antioxidants strategies.

Diseases	Active Ingredient	Nanomaterial	Benefit	Ref.
General wound healing	CaH_2_	Calcium Hydride-Based Dressing	ROS-scavenging, promote wound healing	[47]
	Selenium nanoparticles	BC/Gel/SeNPs hydrogels	Preventing wound infection	[48]
	Nanozyme Cu_2_Se nanosheets	Cu_2_Se/F127 hydrogel	Promoting acute wound healing	[49]
	Curcumin	Chitosan nanoparticles	ROS-scavenging, promote wound healing	[50]
Special wound healing	Ti_3_C_2_ MXene nanosheets	Non-enzymatic antioxidant Mxene	Scavenging ROS, promote infected diabetic wound healing	[51]
	O-nitrobenzene	ZnO@HN hydrogel	Scavenging ROS, promoting infected diabetic wound healing	[52]
	MnO_2_ nanosheets	HMP hydrogel	Antibacterial, reducing inflammation levels, scavenging ROS, promoting infected diabetic wound healing	[53]
	PPY@PDA nanowires	QCS/OD/TOB/PPY@PDA9 hydrogel	Scavenging ROS, promoting infected burn wound healing	[54]
	Curcumin, Mg^2+^	Cur-Mg@PP hydrogel	Anti-inflammatory, antioxidant, promotes burn wound healing	[55]
